# Intrapancreatic Parenchymal Injection of Cells as a Useful Tool for Allowing a Small Number of Proliferative Cells to Grow In Vivo

**DOI:** 10.3390/ijms18081678

**Published:** 2017-08-02

**Authors:** Masahiro Sato, Issei Saitoh, Tomoya Murakami, Naoko Kubota, Shingo Nakamura, Satoshi Watanabe, Emi Inada

**Affiliations:** 1 Section of Gene Expression Regulation, Frontier Science Research Center, Kagoshima University, Kagoshima 890-8544, Japan; 2 Division of Pediatric Dentistry, Department of Oral Health Sciences, Course for Oral Life Science, Graduate School of Medical and Dental Sciences, Niigata University, Niigata 951-8514, Japan; isaito@dent.niigata-u.ac.jp (I.S.); murakami@dent.niigata-u.ac.jp (T.M.); 3 Department of Pediatric Dentistry, Graduate School of Medical and Dental Sciences, Kagoshima University, Kagoshima 890-8544, Japan; kubonao@dent.kagoshima-u.ac.jp (N.K.); inada@dent.kagoshima-u.ac.jp (E.I.); 4 Division of Biomedical Engineering, National Defense Medical College Research Institute, Saitama 359-8513, Japan; snaka@ndmc.ac.jp; 5 Animal Genome Unit, Division of Animal Breeding and Reproduction Research, Institute of Livestock and Grassland Science, National Agriculture and Food Research Organization, Ibaraki 305-0901, Japan; kettle@affrc.go.jp

**Keywords:** cell transplantation, pancreas, iPS cells, ES cells, nude mouse, in vivo cell propagation, tumor cells, solid tumor

## Abstract

In vivo inoculation of cells such as tumor cells and induced pluripotent stem (iPS)/embryonic stem (ES) cells into immunocompromised mice has been considered as a powerful technique to evaluate their potential to proliferate or differentiate into various cell types originating from three germ cell layers. Subcutaneous grafting and grafting under the kidney capsule have been widely used for this purpose, but there are some demerits such as the requirement of a large number of tumor cells for inoculation and frequent failure of tumorigenesis. Therefore, grafting into other sites has been explored, including intratesticular or intramuscular grafting as well as grafting into the cochleae, liver, or salivary glands. In this study, we found that intrapancreatic parenchymal injection of cells is useful for allowing a small number of cells (~15 × 10^3^ cells or ~30 cell clumps μL^−1^·site^−1^) to proliferate and sometimes differentiate into various types of cells. It requires only surgical exposure of the pancreas over the dorsal skin and subsequent injection of cells towards the pancreatic parenchyma under dissecting microscope-based observation using a mouthpiece-controlled glass micropipette. We now name this technology “intrapancreatic parenchymal cell transplantation (IPPCT)”, which will be useful, especially when only a small number of cells or colonies are available.

## 1. Introduction

Induced pluripotent stem (iPS)/embryonic stem (ES) cells have the potential to differentiate into fully differentiated cells originating from three germ layers when they are placed under differentiation-inducing conditions [1,2]. Because of their pluripotential ability, they are thought to be a powerful tool in cell-based therapy to cure damaged tissues in the field of regenerative medicine, although their tumorigenic potential has been a significant challenge for clinical use [3]. To assess the presence of possibly a few remaining undifferentiated iPS/ES cells after inducing their differentiation, inoculation of the differentiated cells into immunocompromised mice, such as nude and non-obese diabetic/severe combined immunodeficient (NOD/SCID) mice, has been considered as a promising in vivo assay, and is also known as “in vivo teratoma formation assay” [4,5,6,7]. When iPS/ES cells are transplanted into immunodeficient mice at growth-permissive sites, they often generate solid tumors called “teratoma” containing various types of differentiated cells [1,2,8,9,10]. Knowing whether the generated teratomas contain differentiated cells from all three germ layers is essential to define the pluripotency of iPS/ES cells [4]. Therefore, the in vivo teratoma formation assay has at least two important aspects, which are the assessment of cell pluripotency and the evaluation of the tumorigenic potential of iPS/ES cell-derived progeny.

For in vivo teratoma formation assays, sites suitable for transplantation of iPS/ES cells are an important factor affecting proper growth of tumor cells. In the past, subcutaneous grafting and grafting underneath the renal capsule have been most widely used [11,12,13,14,15,16,17,18]. However, there are some demerits such as the requirement of a large number of tumor cells for inoculation and frequent failure of tumorigenesis, probably due to the spreading out of inoculated cells [12]. Therefore, grafting into other sites has been explored, including intratesticular [14,19,20,21,22,23], intramyocardial [24], or intramuscular [24,25,26,27,28,29] grafting as well as grafting into the cochleae [30], liver parenchyma [11], or salivary glands [31].

The pancreas is composed of many compartments that are clonally developed and include exocrine acinar cells and endocrine islet cells [32]. It is an organ that is easily accessible surgically, since it is easily exposed over the back skin after surgical dissection of the skin. We previously demonstrated that successful gene delivery was possible when intraparenchymal injection of a plasmid DNA-containing solution was performed using a mouthpiece-controlled glass micropipette, and, subsequently, the injected portion was subjected to in vivo electroporation using tweezer-type electrodes [33]. At that time, we observed that the injected solution remained at the injection site. This means that cells or cell aggregates inoculated within the pancreatic compartment might not spread easily beyond the compartment. In this study, we transplanted actively proliferating tumor cells (including iPS cells) into the pancreatic parenchyma using a mouthpiece-controlled glass micropipette under observation using a dissecting microscope to test whether these cells could grow as solid tumors in vivo. We named this new technology “intrapancreatic parenchymal cell transplantation (IPPCT)”.

## 2. Results

### 2.1. Cells Transplanted into the Pancreatic Parenchyma Are Trapped within Compartments of the Pancreas

**Figure 1:**
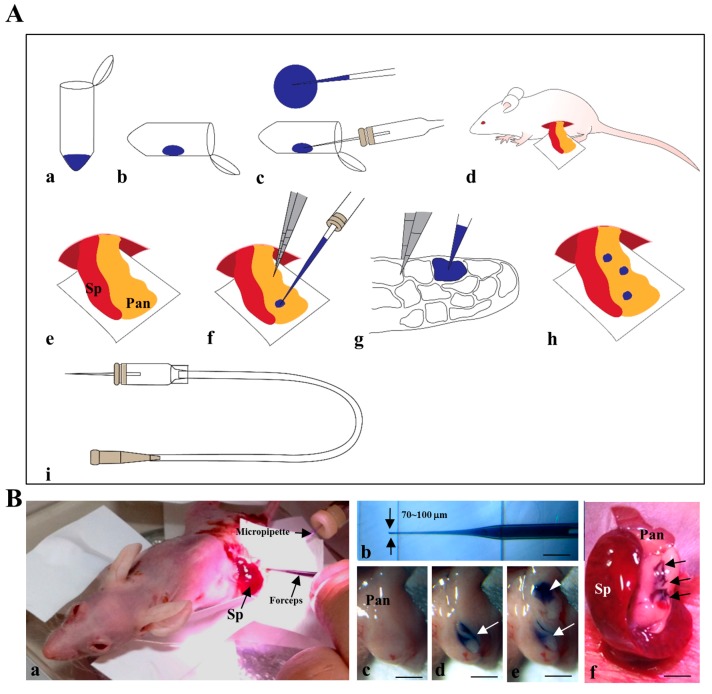
(**A**) Outline of intrapancreatic parenchymal cell transplantation (IPPCT) shown schematically. (**a**–**c**) Sucking ~3 μL of the solution is done by an injection micropipette connected to the mouthpiece; (**i**) under a dissecting microscope. (**d**) The spleen (Sp) and pancreas (Pan) are pulled out after making a small incision on the left dorsal skin of an anesthetized mouse. (**e**–**g**) Approximately 1 μL of the solution is injected by inserting the micropipette into the pancreatic parenchyma. (**h**) Performing a total of three injections at different portions of each pancreas; (**B**) Photographs showing the actual IPPCT procedure. (**a**) Injection towards pancreas under a dissecting microscope. (**b**) Injection micropipette used. (**c**–**e**) The process of IPPCT (before injection **c**, after the 1st injection **d**, and after the 2nd injection **e**). (**f**) The photograph of a pancreas after the 2nd injection. Sp, spleen; Pan, pancreas. Bar = 4 mm.

The IPPCT procedure is schematically illustrated in Figure 1A and will be explained in detail in Section 4.4 “IPPCT” of “Materials and Methods”.

Figure 1B shows the actual process of IPPCT. Briefly, the exposed pancreas of an anesthetized nude mouse was subjected to cell transplantation under a dissecting microscope (Figure 1(Ba)) using a mouthpiece-controlled micropipette (Figure 1(Bb)). In Figure 1(Bc–e), injection was performed twice at each site of the pancreas and can be easily visualized by the color change from pale white before injection (Figure 1(Bc)) to blue after the first injection (Figure 1(Bd), arrow) and after the second injection (Figure 1(Be), arrowheads). The arrows in Figure 1(Bf) show that a total of three sites were successfully injected. Notably, the injected solution was not spread outside the injection site (Figure 1(Bd–f)), suggesting that transplanted cells were confined within several compartments of the pancreas and may not drop out from the site.

**Figure 2:**
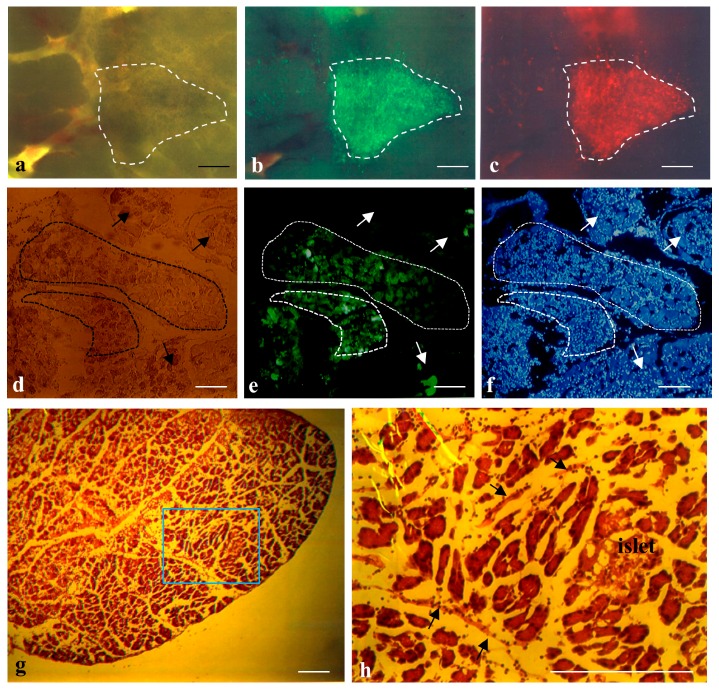
(**a**–**c**) IPPCT using THEPNBS cells carrying tdTomato cDNA (T), hygromycin phosphotransferase gene (H), enhanced green fluorescent protein (EGFP) cDNA (E), puromycin-*N*-acetyltransferase gene (P), neomycin resistance gene (N), blasticidin S deaminase gene (B) and Sh ble gene (S). The THEPNBS cells one day after grafting are enclosed by dotted lines; (**a**) Photograph taken under light; (**b**,**c**) Photographs taken under UV + light. Bar = 40 μm; (**d**–**f**) Cryostat sections of pancreas after intrapancreatic parenchymal gene transfer (IPPGT) using pEGFP-N1. The area surrounded by dotted lines shows compartments containing transfected pancreatic cells expressing EGFP (enhanced green fluorescent protein) strongly. On the other hand, arrows indicate compartments containing non-transfected cells; (**d**) Photograph taken under light; (**e**,**f**) Photographs taken under UV + light. Bar = 30 μm; (**g**,**h**) Cryostat sections of normal pancreas after staining with hematoxylin-eosin; (**h**) An enlarged photography from the quadrant shown in (**g**). Arrows indicate “barrier” separating each compartment. Bar = 2 mm in (**g**) and 200 μm in (**h**).

To confirm this possibility, THEPNBS cells (~10^4^), a porcine embryonic fibroblastic line showing both tandem dimer Tomato (tdTomato)-derived red and enhanced green fluorescent protein (EGFP)-derived green fluorescence simultaneously [34], were injected into the pancreatic parenchyma of adult B6C3F1 mice. The day after inoculation, the pancreas was dissected and then the injection site, which can be easily discernible by the presence of trypan blue, was inspected under a fluorescence dissecting microscope. As expected, both types of fluorescence were detected in the injection site without noticeable cell escape (Figure 2a–c). Intraparenchymal injection of plasmid DNA and subsequent in vivo electroporation [33] can also show how the injected substance is localized within the pancreas. An EGFP-expressing plasmid pEGFP-N1 (0.5–2 μL·site^−1^) was introduced into the pancreatic parenchymal cells via the above method, and the day after the injection site was dissected, fixed, and subjected to cryostat sectioning. The cryostat sections demonstrated that groups of green fluorescent cells were observed in some of the compartments (enclosed by dotted lines in Figure 2d–f), while fluorescent cells were absent in the neighboring compartments (shown by arrows in Figure 2d–f). These findings indicate that the substance injected intraparenchymally remained at the injection site and did not easily diffuse outside of the compartment of the pancreas. Detailed examination of H-E-stained cryostat sections of a normal pancreas demonstrated the presence of barriers (arrows in Figure 2h) that separated one compartment from another, and may have hampered massive diffusion of substances introduced beyond the barriers.

### 2.2. Formation of Solid Tumors after Intrapancreatic Inoculation of Proliferative Cells in Mice

**Figure 3:**
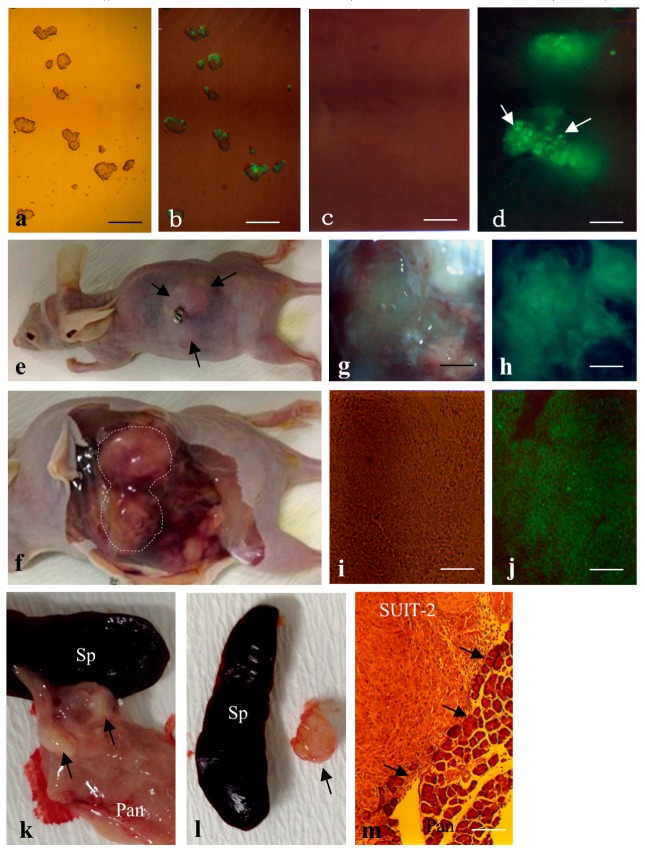
(**a**,**b**) Small clumps of EGFP-F9 just before grafting. Note the presence of distinct EGFP fluorescence in those clumps, although its expression appears mosaic. Bar = 50 μm; (**c**,**d**) Clumps of EGFP-F9 cells one day after IPPCT. Note the presence of distinct fluorescence (arrows in **d**) in the grafted cells. Bar = 50 μm; (**e,f**) Nude mice carrying EGFP-F9 cell-derived solid tumors; (**e**) Before dissection, a mole of solid tumor (arrows) is discernible on the left back; (**f**) Upon autopsy, the enlarged solid tumor (enclosed by dotted lines) is noticeable at the pancreas; (**g**–**j**) A part of the surface area in the dissected solid tumor. Note the presence of distinct fluorescence in that area (shown in (**g**,**h**)). Cryostat section of the fluorescent area shown in (**g**,**h**) indicates the presence of uniform and distinct fluorescence in the EGFP-F9 cell-derived solid tumor (shown in (**i**,**j**)). Bar = 100 μm in (**g**,**h**) and 50 μm in (**i**,**j**); (**k**–**m**) human pancreatic carcinoma cell line SUIT-2 cells 1.5 months after IPPCT. A small solid tumor (arrows in (**k**)) is discernible in the pancreas (Pan); In (**l**), the isolated solid tumor (arrow) is shown with the spleen (Sp). Cryostat section of the dissected SUIT-2-derived solid tumor shows uniform morphology of SUIT-2 cells that are just invading into normal pancreas (Pan), as shown by arrows in (**m**). Bar = 50 μm. (**a**,**c**,**e**–**g**,**i**,**k**–**m**) Photographs taken under light. (**b**,**d**,**h**,**j**) Photographs taken under UV + light.

To test whether a small number of proliferative tumor cells transplanted into the pancreas can proliferate and finally form solid tumors, we first inoculated F9 mouse teratocarcinoma cells [35] that have been engineered to overexpress EGFP under a systemic promoter. Before the inoculation, these cells were induced to form embryoid bodies by culturing on a bacteriological dish for two days (Figure 3a,b). Approximately ten embryoid bodies per injection site (a total of ~30 embryoid bodies/pancreas) were injected into the pancreatic parenchyma of a nude mouse according to the method described in Figure 1A. To check whether the inoculated F9 cells were present in the injection site of the pancreas, mice were sacrificed one day after inoculation, and the injection site in the pancreas was subjected to observation for fluorescence using a fluorescence microscope. As expected, clumps of cells expressing green fluorescence were discernible (Figure 3c,d). When the grafted nude mice were inspected 1.5 months after transplantation, all (2/2) of the nude mice had solid tumors of ~1.5 to 2 cm^3^ in size (Figure 3e,f). A part of these tumors (especially in the internal portion) was necrotic and lost green fluorescence, but the other part, especially near the surface, was intact and fluorescent (Figure 3g,h). Cryostat sectioning of these intact tissues demonstrated that these tumors exhibited homogeneous and undifferentiated cell populations (Figure 3i) reflecting nullipotency of F9 cells themselves and expression of EGFP-derived green fluorescence (Figure 3j).

Similar treatment was performed using the human pancreatic carcinoma cell line SUIT-2 [36]. In this case, ~5 × 10^3^ cells·μL^−1^·site^−1^ were injected. Inspection 1.5 months after inoculation revealed that all (2/2) of the treated mice had distinct solid tumors in their pancreas (arrows in Figure 3k,l). Histological analysis of cryostat sections demonstrated that these tumors were composed of homogeneous cell populations with surrounding normal pancreatic cells (Figure 3m).

**Figure 4:**
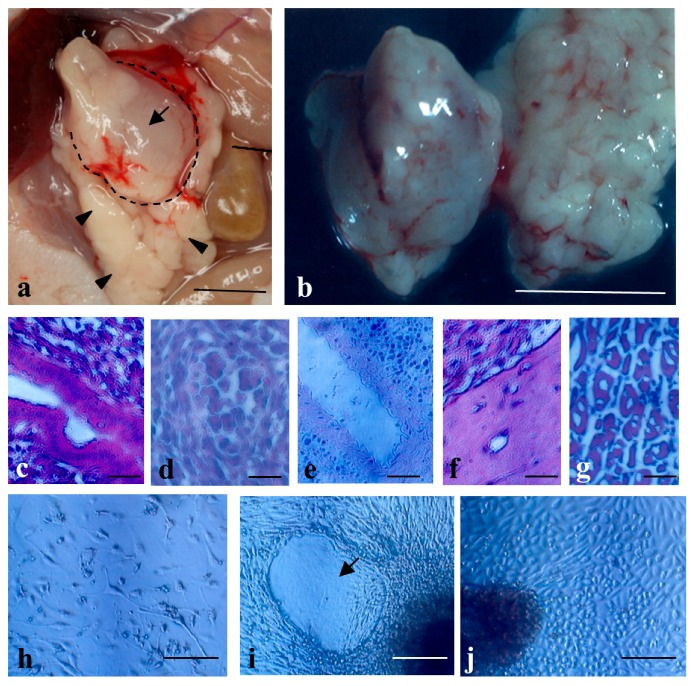
The solid tumors before dissection **a** and after dissection **b** generated 1.5 months after IPPCT using human induced pluripotent stem (iPS) cells (MM-iPS). Note the presence of a solid tumor (arrow in (**a**); also enclosed by dotted lines) generating in the pancreas (arrowheads in (**a**)), Bar = 10 mm; (**c**–**g**) Cryostat sections of the dissected MM-iPS-derived solid tumors showing differentiation of various types of cells, including digestive epithelial cells (**c**), immature blood cells (**d**), tubular structure (**e**), cartilage (**f**), and skeletal muscle (**g**). Bar = 50 μm; (**h**–**j**) Primary culture of cells dissected from a solid tumor shown in (**b**). Note the presence of several types of differentiated cells such as migratory fibroblastic cells (**h**), amorphous structure (arrow in (**i**)), and cuboidal cells like parietal endodermal cells (**j**). Bar = 100 μm.

Next, we transplanted human iPS cells human iPS cells (MM-iPS) (~5 × 10^3^ cells·μL^−1^·site^−1^; [37]). As a result, 83% (5/6) of the mice had distinct teratomas of ~1 cm^3^ in size in their pancreas (Figure 4a,b). There were no appreciable teratomas in the other major organs such as the kidney, heart, intestine, lung, and spleen (data not shown). Histological analysis of cryostat sections demonstrated that these tumors were composed of different tissues/cells originating from three germ layers, including intestinal epithelia (Figure 4c), primitive blood cells (Figure 4d), tubular structure (Figure 4e), cartilage (Figure 4f), and skeletal muscle (Figure 4g). Primary cultivation of the resulting solid teratomas demonstrated the presence of various types of differentiated cells such as migratory fibroblastic cells (Figure 4h), amorphous structure (arrow in Figure 4i), and cuboidal cells like parietal endodermal cells (Figure 4j), suggesting that they had been derived from teratomas.

## 3. Discussion

Teratoma assays are useful for checking the differentiation potentiality of the iPS/ES cells produced. Particularly, when the clinical use of these cells is purposed, the assay may be strictly required in view of transplantation safety. To date, subcutaneous transplantation of iPS/ES cells or their transplantation beneath the kidney capsule into immunocompromised mice, such as nude or NOD/SCID mice, has been widely employed for this purpose. However, a large number of cells (at least >10^6^) is generally required for this purpose, and the grafting rate is not always 100% since the grafted cells are eventually lost, probably due to gradual spreading outside of the injection site [12]. Prokhorova et al. [12] first demonstrated that the formation of human ES cell-derived teratomas was enhanced by the presence of Matrigel, which may inhibit cells from spreading to the outside and is helpful for in vivo expansion of a small number of cells. Since then, several successful reports on teratoma formation have been provided using Matrigel [23,24]. On the other hand, Hentze et al. [29] suggested the usefulness of mixing feeder cells upon grafting of ES cells to increase the efficiency of teratoma formation. Notably, Gropp et al. [17] demonstrated that inoculation of human ES cells (1 × 10^6^) and feeder cells embedded in Matrigel beneath the skin of immunodeficient mice led to 100% generation of teratomas. They also demonstrated that 100 ES cells are enough to grow under this in vivo condition. Our present approach, which allows proliferative cells to grow within compartments of the pancreas, may be in agreement with the concept of Prokhorova et al. [12], since cells grafted into the pancreatic parenchyma rarely escape from the injection site (see Figure 2a–c and Figure 3c,d). In this context, IPPCT with iPS/ES cells plus other factors such as feeder cells and Matrigel may facilitate efficient growth and differentiation of those cells.

In this study, we succeeded in forming solid tumors from ~15 × 10^3^ cells/pancreas using human iPS cells (MM-iPS) and human pancreatic carcinoma cells (SUIT-2) and from ~30 embryoid bodies/pancreas using mouse teratocarcinoma cells (EGFP-F9) in the pancreas of nude mice (BALB/c-nu/nu). In general, more than 1 × 10^6^ iPS/ES cells are required for teratoma formation assay using subcutaneous transplantation and cell grafting beneath the renal capsule [14], although approximately 1 × 10^5^ cells for intramyocardial grafting [24] and 1 × 10^4^ cells for intramuscular grafting [24] have been reported to be the minimum number of cells needed to form teratomas. In this context, our system may be useful, especially when only a small number of cells or colonies are available.

Notably, similar methods allowing in vivo growth of tumor cells in pancreatic parenchyma have been described by Kim et al. [38] and Jiang et al. [39]. The former group injected 5 × 10^5^ tumor cells suspended in a 100 μL volume using a 25-guage needle, and observed no gross leakage from the injection site. The latter group injected 1 × 10^6^ tumor cells suspended in a 20 μL volume using a 27-guage needle, and observed leakage of an injected solution after the needle was pulled out. In each case, successful tumorigenesis has been achieved by injection of a large number of tumor cells, which is clearly in contrast with our present approach, based on injection of a small number of tumor cells.

The concept that the graft site may influence teratoma formation efficiency or the differentiation potential of iPS/ES cells is an important issue to be considered, as suggested by Cooke et al. [11] and Prokhorova et al. [12]. For example, Hara et al. [40] demonstrated that human ES cells transplanted into mouse retina were induced to exhibit neural differentiation. However, others have found little or no effect [14]. In our case, human iPS cells grafted into the pancreatic parenchyma exhibited differentiation of various types of cells (see Figure 4c–g), suggesting that the pancreas may be one of the organs having little or no effect on the differentiation potential of iPS/ES cells.

This present system appears also to be useful for testing the ability of therapeutic cells to cure diseases such as type 1 diabetes. This may be exemplified in the field of regenerative medicine by grafting mesenchymal stem cells that have the potential to produce functional insulin. Interestingly, Yanai et al. [41] demonstrated that grafting of fused cells between mesenchymal stem cells and islet cells under the renal capsule of diabetic animals caused decreased levels of blood glucose for a long period. Our system allows the survival of a small number of cells after IPPCT. In this context, autologous grafting of the fused cells derived from individuals or cells that are engineered to express active insulin into the pancreas of the NOD mice, a polygenic model for autoimmune type 1 diabetes, may be of interest for future trials.

## 4. Materials and Methods

### 4.1. Animals

All animal experiments were performed in agreement with the guidelines of Kagoshima University Committee on Recombinant DNA Security and approved by the Animal Care and Experimentation Committee of Kagoshima University (permit no. 25035 and 25036; dated 8 August 2013). All surgeries were performed under three anesthetics (medetomidine, midazolam, and butorphanol), and all efforts were made to minimize suffering. For the inoculation of tumor cells into the pancreas, eight- to twenty-week-old immunodeficient female mice (BALB/c-nu/nu, CLEA Japan Ltd., Tokyo, Japan) were used. In some cases, adult female B6C3F1 (a hybrid between B6 and C3H; CLEA Japan Ltd., Tokyo, Japan) were used.

### 4.2. Cell Culture 

Cells termed MM-iPS, which are derived from human deciduous dental pulp cells, were used as a source of multipotential human iPS cells. EGFP-F9 cells, a stable F9 teratocarcinoma cell line expressing EGFP cDNA obtained by transfection with pEGFP-N1 plasmid (Invitrogen Co., Carlsbad, CA, USA) and subsequent selection with G418, were used as a source of nullipotential murine teratocarcinoma cells. SUIT-2 cells, taken from a cell line isolated from human pancreatic carcinoma, were used as a source of malignant human tumor cells. THEPNBS cells, which are porcine embryonic fibroblastic cells that have been engineered to express both tdTomato and EGFP-derived fluorescence, were used as a source of fluorescent normal cells.

MM-iPS cells were maintained on mytomycin C-treated (#M4287; Sigma-Aldrich Co., Ltd., St. Louis, MO, USA) mouse embryonic feeder cells in a 60-mm gelatin-coated dish (#4010-020; Iwaki Glass Co., Ltd., Tokyo, Japan) with human ES cell culture medium iPSellon (#007001; Cardio, Kobe, Japan) supplemented with 5 ng/mL recombinant human basic fibroblast growth factor (#064-04541; Wako Pure Chemical Industries Ltd., Osaka, Japan). EGFP-F9 and SUIT-2 cells were grown in Dulbecco’s modified Eagle’s medium (DMEM) containing high glucose (#11995; Invitrogen Co., Carlsbad, CA, USA) with 10% fetal bovine serum (FBS) and 1% antibiotic-antimycotic solution (#A5955; Sigma-Aldrich Co., Ltd., St. Louis, MO, USA) THEPNBS cells were grown in culture medium consisting of DMEM/Ham’s F-12 (#124; Wako Pure Chemical Industries Ltd., Osaka, Japan), 10% FBS, and 1% antibiotic-antimycotic solution. All cells were maintained at 37°C in an atmosphere of 5% CO_2_ in air.

Just prior to inoculation of tumor cells into the pancreas, MM-iPS, SUIT-2, and THEPNBS cells were harvested by trypsinization and suspended in Dulbecco’s Ca^2+^-Mg^2+^-free phosphate buffered saline (D-PBS(−)), pH 7.4 (#14249-95; Nacalai Tesque, Inc., Tokyo, Japan). After calculating the number of viable cells using a disposable 4-chamber hemocytometer (#521-10; Funakoshi Co., Ltd., Tokyo, Japan), MM-iPS, SUIT-2, and THEPNBS cells were diluted to ~5 × 10^3^ cells·μL^−1^ in 20 μL of D-PBS(−) and 0.1% (*v*/*v*) trypan blue (Trypan Blue Stain 0.4%; Invitrogen Co., Carlsbad, CA, USA) in a 1.5 mL tube. In the case of inoculating EGFP-F9 cells into the pancreas, the cells were subjected to embryoid body formation since they are easily identifiable when the grafted tissue is inspected under an Olympus BX60 microscope. Briefly, after trypsinization, 1 × 10^5^ EGFP-F9 cells were transferred onto a 30 mm bacteriological dish (#1000-035; Iwaki, Tokyo, Japan) with normal medium used for EGFP-F9 and maintained for two days. During suspension culture, small embryoid bodies (10–30 cells per clump) were formed. At that time, they were collected and diluted to ~10 embryoid bodies·μL^−1^ in 20 μL of D-PBS(−) and 0.1% (*v*/*v*) trypan blue in a 1.5 mL tube.

In the case of primary cultivation from iPS cell-derived solid tumors grown in the pancreas, the dissected tumor was minced by sterile scissors on the surface of a 30 mm tissue culture dish (#3000-035; Iwaki Co., Ltd., Tokyo, Japan) in aseptic conditions and finally dissolved in 3 mL of D-PBS(−). The cell suspension was moved into a 15-mL conical tube (#11-039-002; Iwaki Co., Ltd., Tokyo, Japan) and left for 10 min at room temperature to sediment larger clumps. Next, the supernatant was picked up by a 1-ML pipette tip (Ina Optika Co., Ltd., Osaka, Japan) and subjected to brief centrifugation to obtain single cells. They were then cultivated in iPSellon mentioned above in a 60 mm tissue culture dish (#3010-060; Iwaki Co., Ltd., Tokyo, Japan) to propagate their derivatives. 

### 4.3. Intrapancreatic Parenchymal Gene Transfer (IPPGT)

Gene delivery towards murine pancreatic parenchyma was performed according to the method described by Sato et al. [33] who named this technique “intrapancreatic parenchymal gene transfer (IPPGT).” Prior to this, the glass capillary (#GDC-1, Narishige Scientific Instrument Lab., Tokyo, Japan) was pulled by a micropipette puller (P-97/IVF; Sutter Instrument Company, CA, USA) to make a pointed end. The tip of the micropipette was broken using microscissors (#MB-53, NAPOX, Natsume Co., Ltd., Tokyo, Japan) under a dissecting microscope (SZX10; Olympus, Tokyo, Japan) to make its inner diameter 30 to 50 μm. Approximately 1–2 μL of a solution containing pEGFP-N1 (0.5 μg·μL^−1^) and 0.1% (*v*/*v*) trypan blue marker for visualization of the injected solution in D-PBS(−) was sucked using a micropipette connected to a mouthpiece (Figure 1(Ai)) while watching the droplet being drawn under the SZX10 microscope, as shown schematically in Figure 1(Aa–c). Immediately, the injection site was held by tweezer-type electrodes and subjected to in vivo electroporation using a Nepa21 electroporator (NEPA Co., Ltd., Chiba, Japan). On the next day, the injection site that had been fluorescent owing to the expression of EGFP was dissected under observation using an SZX10 microscope attached to the lamps as an excitation source for fluorescence and immediately fixed in 4% paraformaldehyde (PFA) in D-PBS(−) at 4 °C for two days prior to cryostat sectioning.

### 4.4. IPPCT

Transplantation of cells or cell aggregates into the parenchyma of adult mouse pancreas was performed according to the IPPGT method, except that the substance injected was changed from a plasmid DNA-containing solution to a cell-containing solution. Figure 1A shows the outline of the IPPCT procedure schematically. First, a solution containing cells (or aggregates) and 0.1% (*v*/*v*) trypan blue was transferred to the inner surface of a 1.5 mL tube near the cap by using a 200-μL yellow tip, and then approximately 3 μL of the solution was sucked by an injection micropipette connected to the mouthpiece (shown in Figure 1(Ai)) under observation with a dissecting microscope (Figure 1(Aa–c)). Next, a small incision was made on the left dorsal skin of an anesthetized mouse, and then the spleen and pancreatic tail were pulled out (Figure 1(Ad)). Immediately, this micropipette was inserted into the pancreatic parenchyma under observation with an SZX10 dissecting microscope and approximately 1 μL of the solution was injected (Figure 1(Ae–g)). Correct injection was visibly monitored by a rapid color change (from pale white to blue) at the injection site (Figure 1(Bc–e)). This operation was repeated twice. Thus, a total of three injections were performed at each portion (Figure 1(Ah,Bf).

### 4.5. Histological Analysis

Animals were sacrificed one day or 1.5 months after cell transplantation. For IPPCT using THEPNBS and EGFP-F9, the grafted portions, which are easily detectable with an SZX10 dissecting microscope because of the presence of trypan blue, were dissected one day after grafting and placed on a glass slide. Then, the tissue sample was squashed gently after placing a cover slip prior to observation for fluorescence, described below. The growing solid tumors and surrounding pancreatic tissues were sampled 1.5 months after grafting. The sacrificed animals were also subjected to general inspection for tumor invasion into other organs. A part of the dissected tumor was fixed with 4% PFA in D-PBS(−) at 4 °C for two days, dehydrated by immersion in 0.25% sucrose in D-PBS(−) at 4 °C for two days, and then dehydrated in 0.4% sucrose in PBS at 4 °C for four days. These samples were then embedded in optimum cutting temperature (O.C.T.) compound (Tissue-Tek^®^ [no. 4583]; Miles Scientific, Naperville, IL, USA) for cryostat sectioning. Some cryostat sections were stained with hematoxylin and eosin (H-E), and others were embedded in a solution containing glycerol and 600 nM 4,6-diamidino-2-phenylindole (Molecular Probes Inc., Eugene, OR, USA) for 5 min at room temperature and inspected for fluorescence using a fluorescence microscope (BX60; Olympus, Tokyo, Japan). In some cases, especially for iPS cell-derived solid tumors, a part of the solid tumor was dissected by sterile scissors in aseptic conditions and subjected to primary cultivation as mentioned above.

### 4.6. Fluorescence Observation

Fluorescence in the samples was examined under a fluorescence BX60 microscope using DM505 (BP460-490 and BA510IF; Olympus) and DM600 (BP545-580 and BA6101F; Olympus) filters, which were used to detect EGFP-derived green fluorescence and tdTomato-derived red fluorescence, respectively. The H-E-stained sections were also inspected using a BX60 microscope. Micrographs were taken using a digital camera (FUJIX HC-300/OL; Fuji Film, Tokyo, Japan) attached to the fluorescence microscope, and images were printed using a Mitsubishi digital color printer (CP700DSA; Mitsubishi, Tokyo, Japan).

### 4.7. Quantification of Teratoma Formation

iPS cell-derived teratomas developing in the pancreas were estimated by visual inspection of H-E-stained sections. Differentiated elements in the teratomas were classified into ectodermal (skin, neural, etc.), endodermal (primitive glandular structures, gastrointestinal epithelial cells, etc.), and mesodermal (cartilage, bone, mensenchymal cells, etc.) components.

## 5. Conclusions

We have presented an approach that allows the growth of actively proliferative cells from a small number of cells in vivo. This method is also applicable in in vivo teratoma formation assays, which may be used for the assessment of cell pluripotency and preclinical biosafety analysis of iPS/ES cells.
